# Identification of circRNA-miRNA-mRNA regulatory network and its role in cardiac hypertrophy

**DOI:** 10.1371/journal.pone.0279638

**Published:** 2023-03-23

**Authors:** Ke Gong, Kai Yang, Ting Xie, Yong Luo, Hui Guo, Zhiping Tan, Jinlan Chen, Qin Wu, Yibo Gong, Luyao Wei, Jinwen Luo, Yao Yao, Yifeng Yang, Li Xie

**Affiliations:** 1 Department of Cardiovascular Surgery, The Second Xiangya Hospital of Central South University, Central South University, Changsha, P.R. China; 2 Department of Plastic Surgery, The Second Xiangya Hospital of Central South University, Central South University, Changsha, P.R. China; 3 The Clinical Center for Gene Diagnosis and Therapy of The State Key Laboratory of Medical Genetics, The Second Xiangya Hospital of Central South University, Central South University, Changsha, Hunan, P.R. China; 4 Department of Cardiothoracic Surgery, Hunan Children’s Hospital, Changsha, Hunan, P.R. China; 5 Department of Blood Transfusion, The Second Xiangya Hospital of Central South University, Central South University, Changsha, P.R. China; Indiana University School of Medicine, UNITED STATES

## Abstract

**Background:**

Hypertrophic cardiomyopathy (HCM) is a grave hazard to human health. Circular RNA (circRNAs) and micro RNA (miRNAs), which are competitive endogenous RNA, have been shown to play a critical role inHCM pathogenicity. However, to a great extent, the biological activities of ceRNA in HCM pathophysiology and prognosis remain to be investigated.

**Materials and methods:**

By analyzing the expression files in the Gene Expression Comprehensive (GEO) database, differentially expressed (DE) circRNAs, miRNAs, and mRNAs in HCM were identified, and the target molecules of circRNAs and miRNAs were predicted. The intersection of the differentially expressed RNA molecules and the expected target was then calculated, and a ceRNA network was subsequently constructed using RNA molecules. Using Gene Ontology (GO) and Kyoto Encyclopedia of Genes and Genomes (KEGG) pathway analyses, the potential etiology was elucidated. qPCR was used to validate a portion of the hub gene using Angiotensin II to generate a cell hypertrophy model.

**Results:**

Three large-scale HCM sample datasets were extracted from the GEO database. After crossing these molecules with their expected targets, the circRNA-miRNA-mRNA network had two DEcircRNAs, two DEmiRNAs, and thirty DEmRNAs, compared to normal tissues. Functional enrichment analysis of GO and KEGG demonstrated that many of the HCM pathways and mechanisms were associated with calcium channel release, which is also the primary focus of future research. The qPCR results revealed that circRNA, miRNA, and mRNA expression levels were different. They may include novel noninvasive indicators for the early screening and prognostic prediction of HCM.

**Conclusion:**

In this study, we hypothesized a circRNA-miRNA-mRNA regulation network that is closely related to the progression and clinical outcomes of HCM and may contain promising biomarkers and treatment targets for HCM.

## Introduction

Hypertrophic cardiomyopathy (HCM) is one of the most prevalent hereditary heart illnesses, affecting approximately 0.2% of the world’s population (1 in 500) [1]. HCM is characterized by an increase in ventricular wall thickness. Potential HCM causes include over 1,500 mutations in at least 15 genes encoding cardiac sarcomere-associated protein components [2–4]. MYH7 and MYBPC3 code for the myosin heavy chain and myosin binding protein C, respectively, and represent 50–60% of the HCM gene family [5]. However, there are still a significant number of patients whose hereditary genes have not been identified, highlighting the need for precision therapy, given the HCM genetic diversity [6–8]. The leading causes of death include sudden death, heart failure, and thromboembolism, which account for one percent of the annual global mortality rate [9]. HCM is a common cause of unexpected death in young adults [10]. However, the majority of affected individuals may remain unidentified, and many will not experience a significant reduction in life expectancy or significant symptoms [11].

A difficult aspect of identifying HCM is the lack of association between genotype and phenotype, as members of the same family with the same mutation exhibit distinct symptoms [12]. Given its various clinical presentations, phenotypic heterogeneity, vast number of mutations, and substantial consequences, HCM is considered a highly complex illness [13]. HCM may be caused by a mixture of endogenous gene mutations, exogenous protein-protective mechanisms, and environmental variables [2, 4, 14]. Consequently, it is crucial to identify the epigenetic alterations that may initiate HCM, particularly the recently identified hot spot circular RNA (circRNA) [15].

In recent years, circRNA and microRNAs (miRNAs), which are non-coding RNAs, have received a great deal of interest for their role in a variety of disorders. These RNA molecules modulate gene expression through intricate connections and processes. circRNAs are covalently closed endogenous biomolecules in eukaryotes with tissue- and cell-specific expression patterns. Their synthesis is regulated by unique cis-acting elements and trans-acting factors. Some circRNAs are numerous and are evolutionarily stable. Numerous circRNAs perform vital biological functions by acting as miRNA or protein inhibitors ("sponges"), controlling protein functions, or translating themselves. circRNA is also associated with diabetes, neurological illnesses, cardiovascular diseases, and cancer [16, 17]. Competitive endogenous RNAs (ceRNAs) possess sufficient miRNA response elements to bind to and compete with matching miRNAs, isolating them at the post-translational stage and controlling mRNA production [18]. miRNAs are short noncoding RNA consisting of 20–22 nucleotides. They regulate gene expression by lowering messenger RNA stability in various normal and pathological processes [19, 20]. Consequently, the ceRNA regulatory network, comprising circRNA-miRNA-mRNA, plays an essential role in disease regulation.

In this study, we obtained the expression patterns of mRNA, miRNAs, and circRNAs from the Comprehensive Gene Expression (GEO) database for patients with HCM. Using R software, differentially expressed (DE) mRNA, miRNAs, and circRNAs were identified. Additionally, circRNA-miRNA and miRNA-mRNA prediction targets were identified. a circRNA-miRNA-mRNA network was then constructed by merging them. Popular enrichment analysis techniques, such as protein-protein interaction (PPI), gene ontology (GO), and Kyoto Encyclopedia of Genes and Genomes (KEGG) analyses, were used to anticipate the potential disease processes and regulatory mechanisms of HCM. This study offers new diagnostic biomarkers, therapeutic targets, and major hints for future research, which may enhance our understanding of the probable molecular processes of HCM. Although hypertrophic cardiomyopathy is mainly caused by genetic factors, the process of myocardial hypertrophy and remodeling is similar to that of myocardial remodeling caused by other factors. Previous studies have used angiotensin II (Ang II) to construct HCM models. Therefore, we also used this drug to build an HCM model to study its regulatory targets. In this study, Ang II was employed to generate a cell hypertrophy model, and a quantitative real-time polymerase chain reaction was used to validate some of the hub genes.

## Materials and methods

### Expression profile data and quality assessment

First, three microarray expression profile datasets from the National Center for Biotechnology Information were selected. Based on the mRNA expression microarray data of GPL15389, Illumina HumanHT-12 V3.0 expression bead chip, GSE36961 contained 145 samples (106 HCM samples and 39 control samples). Based on the GPL8179 and Illumina Human v2 MicroRNA expression bead chip, the GSE36946 dataset included miRNA expression profiles of 107 HCM patients and 20 controls. The GSE148602 dataset contained the circRNA expression profiles of the cardiac tissues of 15 HCM patients and 7 controls using the GPL28387, Agilent-085202 LC human ceRNA array. GEO2R was used to assess the quality control between HCM and normal samples. GEO2R is an online application for comparing and analyzing differentially expressed genes (DEG) in HCM and normal samples using the limma and GEOquery R libraries of the bioconductor project. GEO2R was solely utilized for quality control, and the DEG derived from its analysis was not implemented. We created a histogram of P-values for each gene. S1 File contains all original data.

### Retrieval and screening of array data of differentially expressed mRNA, miRNA, and circRNA in HCM

**Fig 1:**
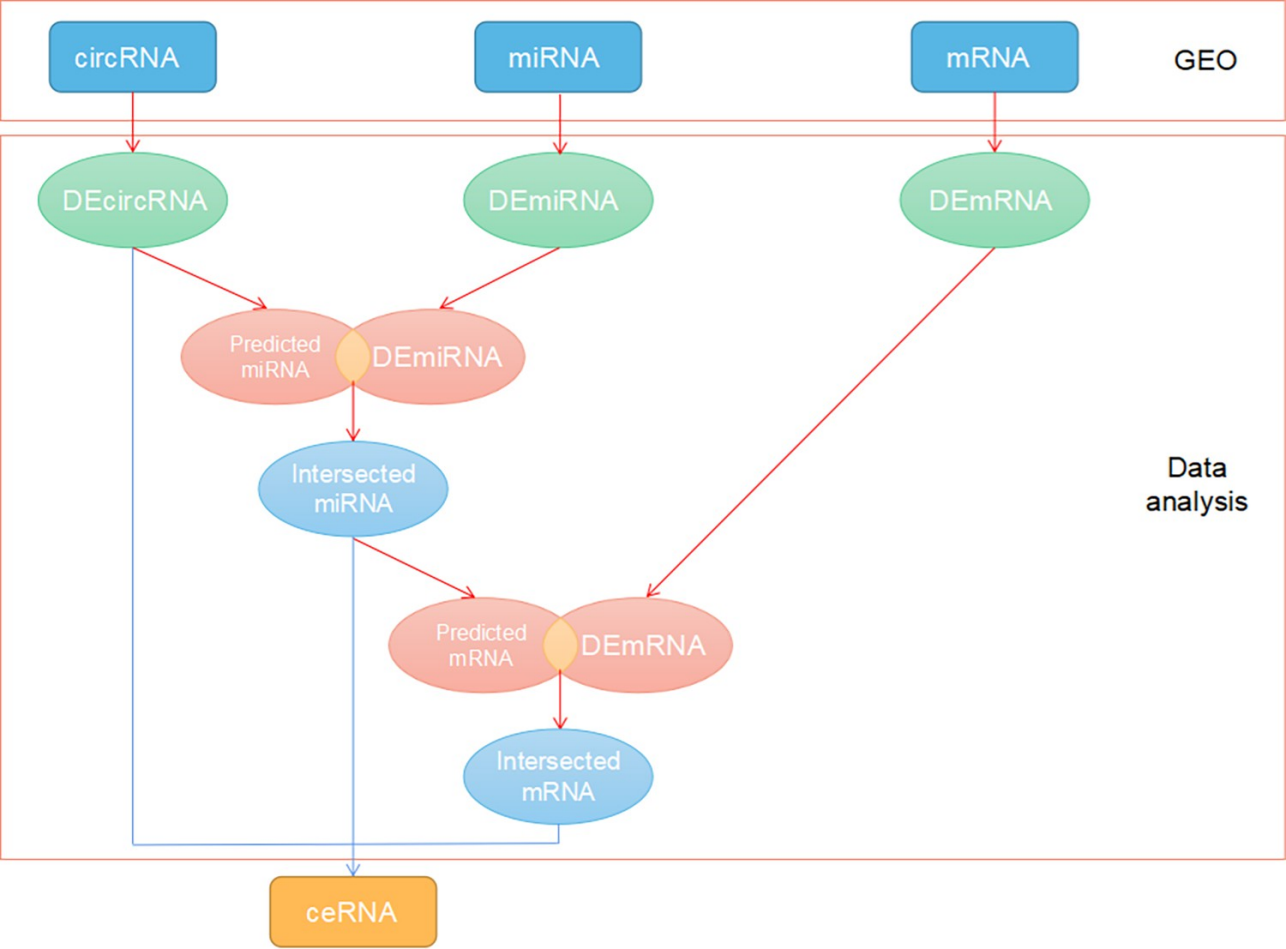
Ideas and flow chart of the entire research.

Perl and R were used for the data analysis and processing [21, 22]. All source codes are contained in the S2 File. Each dataset was annotated and organized using the Perl software. We screened DEmRNAs, DEmiRNAs, and DEcircRNAs using the GSE36961, GSE36946, and GSE148602 datasets and data molecules. The R software "limma" package was used to compare the HCM samples with normal samples. |log2FC|> 2 and P value0.05 were our cut-off criteria for circRNA data determination. We established cutoff standards for miRNA and mRNA as |log2FC>0.5 and P value0.05. The "gplots" software was utilized to generate the volcanic maps and heat maps of DEmRNA, DEmiRNA, and DEcircRNA. Fig 1 displays a flowchart of the entire process.

### Target prediction and crossover constructed by the ceRNA network

In the circBase database, DEcircRNA-specific information can be acquired [23]. RNAhybrid and miRanda were used to predict the combination of circRNAs and miRNAs. Eventually, promising miRNAs were identified after crossing them with DEmiRNAs. mRNA targets were predicted using TargetScan, Starbase, and miRDB databases. When two databases supported mRNA as candidate targets, the final mRNA was obtained by intersecting these mRNA with DEmRNA. Based on the aforementioned findings, a circRNA-miRNA-mRNA network was constructed and visualized using Cytoscape 3.7.2.

### GO and KEGG functional enrichment analysis

To elucidate the pathophysiological process and critical signaling pathways of HCM, the GO and KEGG bioinformatics analysis tools were utilized. The R/Bioconductor program "clusterProfiler" was used to examine GO word and KEGG pathway enrichment. Adjusted P<0.05 was considered the statistical threshold.

### Cell culture

All cells were obtained from Professor Yifeng Yang. The American Type Culture Collection (Manassas, VA, USA) was used to obtain human AC16 cardiomyocytes (ATCC, Manassas, VA). The cells were grown at 37°C in a humidified incubator containing 5% CO2, per the ATCC standards. AC16 cells were cultured in a DMEM/F12 (Thermo Fisher Scientific, Waltham, MA, USA) mixture with 10% FBS (Thermo Fisher Scientific, Waltham, MA, USA) and 1% antibiotics (Thermo Fisher Scientific, Waltham, MA, USA). After 48 h of subculturing, the cells were treated for 24 h with Ang II 10–7 M to induce hypertrophy.

### Western blot (WB)

AC16 cells were eliminated from the cell hypertrophy model. The cells were rinsed with cold phosphate-buffered saline and lysed with radioimmunoprecipitation assay lysis buffer (89901; Thermo Fisher Scientific) containing 1% protease and phosphatase inhibitor (78442; Thermo Fisher Scientific). The lysate protein concentration was determined according to the manufacturer’s instructions using a total protein quantification kit (Thermo Fisher Scientific, 23227). Protein samples (20 μg) were separated by sodium lauryl sulfate-polyacrylamide gel electrophoresis at a concentration of 10% and then transferred to a polyvinylidene fluoride membrane. The membrane was sealed with 5% skim milk for 1.5 h before being incubated overnight at 4°C with the primary antibodies MYH7 (Proteintech, 22280-1-AP) and GAPDH (Proteintech, 10494-1-AP). The following day, following three washes with Tris-buffered saline and Tween 20, the membrane was incubated with IgG secondary antibodies (Abcam, ab97051) at 37°C for 1 h, and then washed thrice with TBST. Antibody-binding proteins were detected using an enhanced chemiluminescence reagent (34580; Thermo Fisher Scientific). ImageJ was used to quantitatively assess the grayscale of each band.

### qPCR

The GeneJET RNA Purification Kit (Thermo Fisher Scientific) was used to extract total RNA from these cell lines according to the manufacturer’s instructions. cDNA was synthesized using the RevertAid First Strand cDNA Synthesis Kit (Thermo Fisher Scientific, USA) according to the manufacturer’s instructions. qPCR was conducted using PowerUpTM SYBRTM Green Master Mix (Thermo Fisher Scientific) on a Thermo 9700 rapid real-time PCR machine. The following primers were used: circRNA h-hsa circ 0079270-F,5’-CAGGATGCAGAAGGAGATCACT-3’; circRNA h-hsa circ 0079270-R,5’-GATATCATCATCCATGGTGAGCTT-3’. RiboBio (RiboBio Co. Ltd, Guangzhou, Guangdong, China) Bulge-loopTM miRNA qRT-PCR Primer Set (one RT primer and a pair of qPCR primers for each set) for hsa-miR-34c-5p and hsa-miR-34c-5p was used to determine the miRNA concentration. Primer sequences for other mRNAs are shown in S3 File. The internal controls for circRNA and miRNA were GAPDH and U6, respectively. RNA fold changes were calculated using the 2−ΔΔCT method. S4 File contains all data.

### Statistical analysis

All information is presented as the mean standard deviation. All statistical analyses were performed using GraphPad Prism 8.0 (GraphPad Software, San Diego, CA, USA). The significance of the differences between the two groups was determined using a two-tailed Student’s t-test. SPSS was used to generate the statistical results. In this investigation, P-values <0.05 were statistically significant unless otherwise specified.

## Results

### Quality control test results and identify differentially expressed and crossover circRNA, miRNA, and mRNA

**Fig 2:**
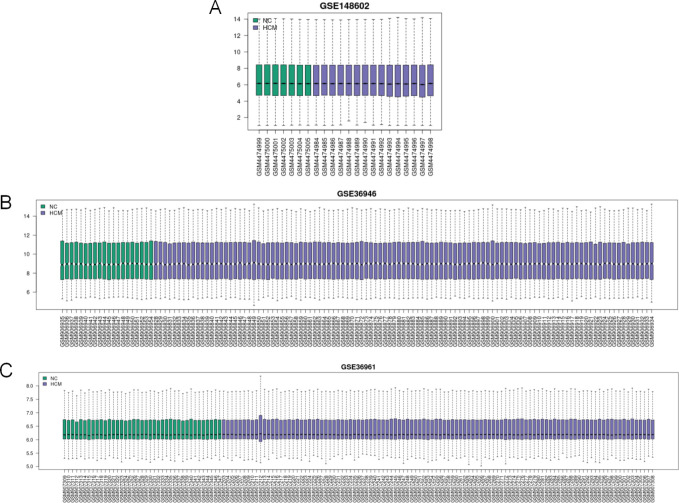
Quality control chart. A, GSE148602; B, GSE36946; C, GSE36961.

**Fig 3:**
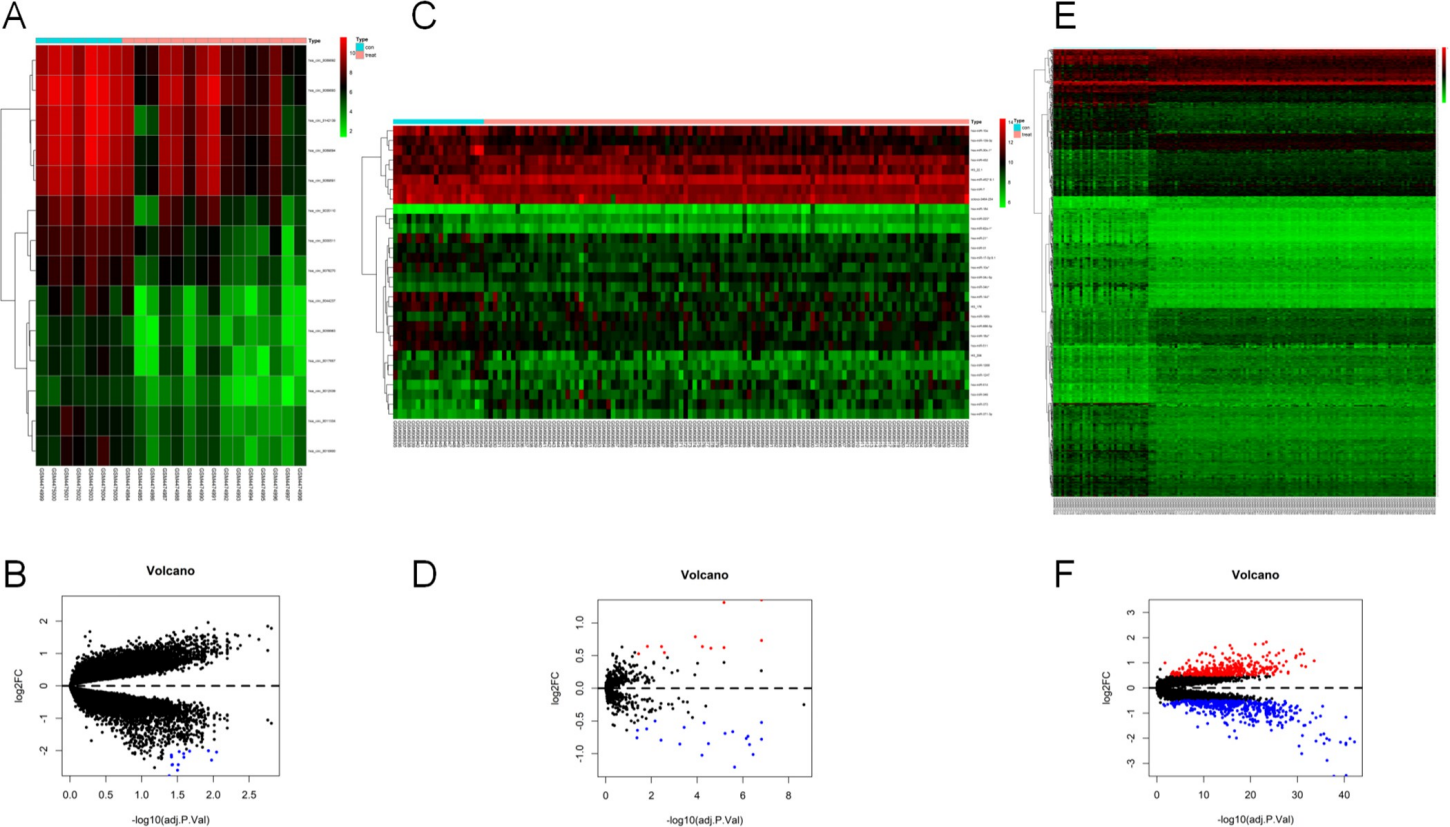
Heatmaps and volcano maps. A and B, GSE148602; C and D, GSE36946; E and F, GSE36961. The heat map shows high expression in red and low expression in green. The volcano map shows high expression in red and low expression in green.

**Fig 4:**
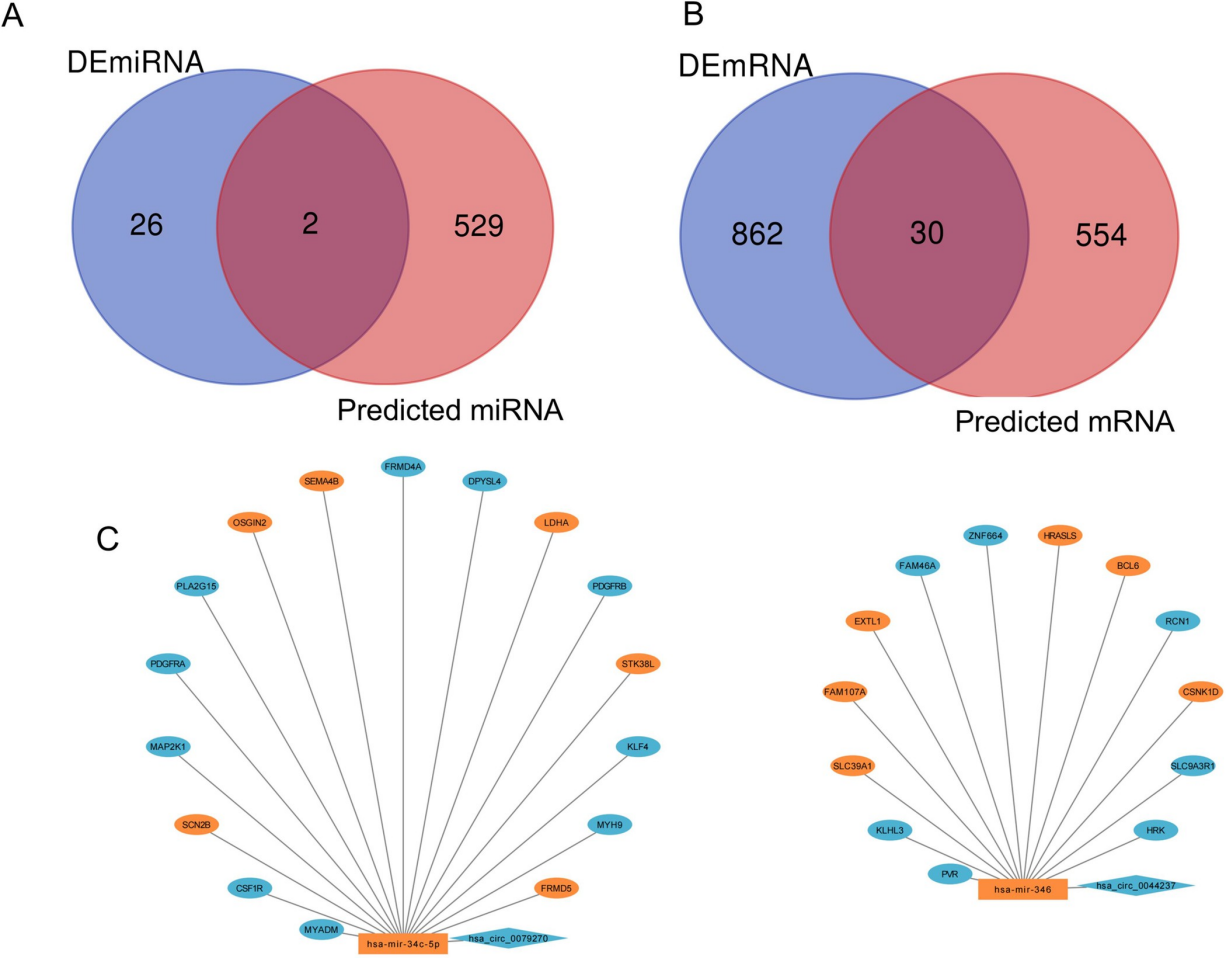
Venn diagram and ceRNA network. A, Intersection of predicted miRNA and DEmiRNA; B, Intersection of predicted mRNA and DEmRNA; C, ceRNA network. The expression levels of these RNA molecules were indicated by different colors, with blue representing low expression and red representing high expression.

The results indicate that there was no significant variation in the distribution of most of the genes between the samples, and the corresponding data are provided (Fig 2). Three GEO datasets (GSE36961, GSE36946, and GSE148602) were obtained from the GEO website. In the circRNA profile data of GSE148602, there were a total of 14 DEcircRNAs between HCM samples and normal controls, and their expression levels were downregulated in HCM cardiac tissues. Using miRanda and RNAhybrid, we predicted that 531 miRNAs could be targets of these 14 circRNAs. For abnormally expressed miRNAs in GSE36946, 28 DEmiRNAs in HCM were analyzed, of which 10 were overexpressed and 18 were downregulated. Two miRNAs were found at the intersection of 531 miRNAs and 28 DEmiRNAs predicted from the miRanda and RNAhybrid databases, respectively. TargetScan, starBase, and miRDB databases identified 584 potential target genes for the two cross miRNAs. In the GSE36961 mRNA expression profile, 892 mRNAs were differentially expressed, with 387 mRNAs being strongly expressed in HCM and 505 mRNAs expressed at lower levels in HCM than in normal tissues. A total of 30 mRNA hybrids were produced. The crossover states of miRNAs and mRNA were illustrated using Venn diagrams, and volcano maps and heatmaps were created for GSE36961, GSE36946, and GSE148601, respectively (Figs 3, 4A and 4B).

### Construction of HCM-specific ceRNA network

Based on the two circRNAs (hsa_circ_0079270 and hsa_circ_0044237), two miRNAs (hsa-miR-34c-5p and hsa-miR-346), and 30 mRNAs (HRK, FRMD5, MYH9, SLC9A3R1, KLF4, STK38L, PDGFRB, LDHA, DPYSL4, CSNK1D, RCN1, BCL6, FRMD4A, SEMA4B, OSGIN2, HRASLS, ZNF664, PLA2G15, NFE2L1, EXTL1, PTPRM, PDGFRA, FAM107A, MAP2K1, SLC39A1, SCN2B, CSF1R, KLHL3, PVR, and MYADM), Cytoscape 3.7.2 was used to design a circRNA-miRNA-mRNA regulatory network. Different hues represent the expression levels of these RNA molecules, with blue signifying low expression and red signifying high expression (Fig 4C).

### GO and KEGG function enrichment analysis

**Fig 5:**
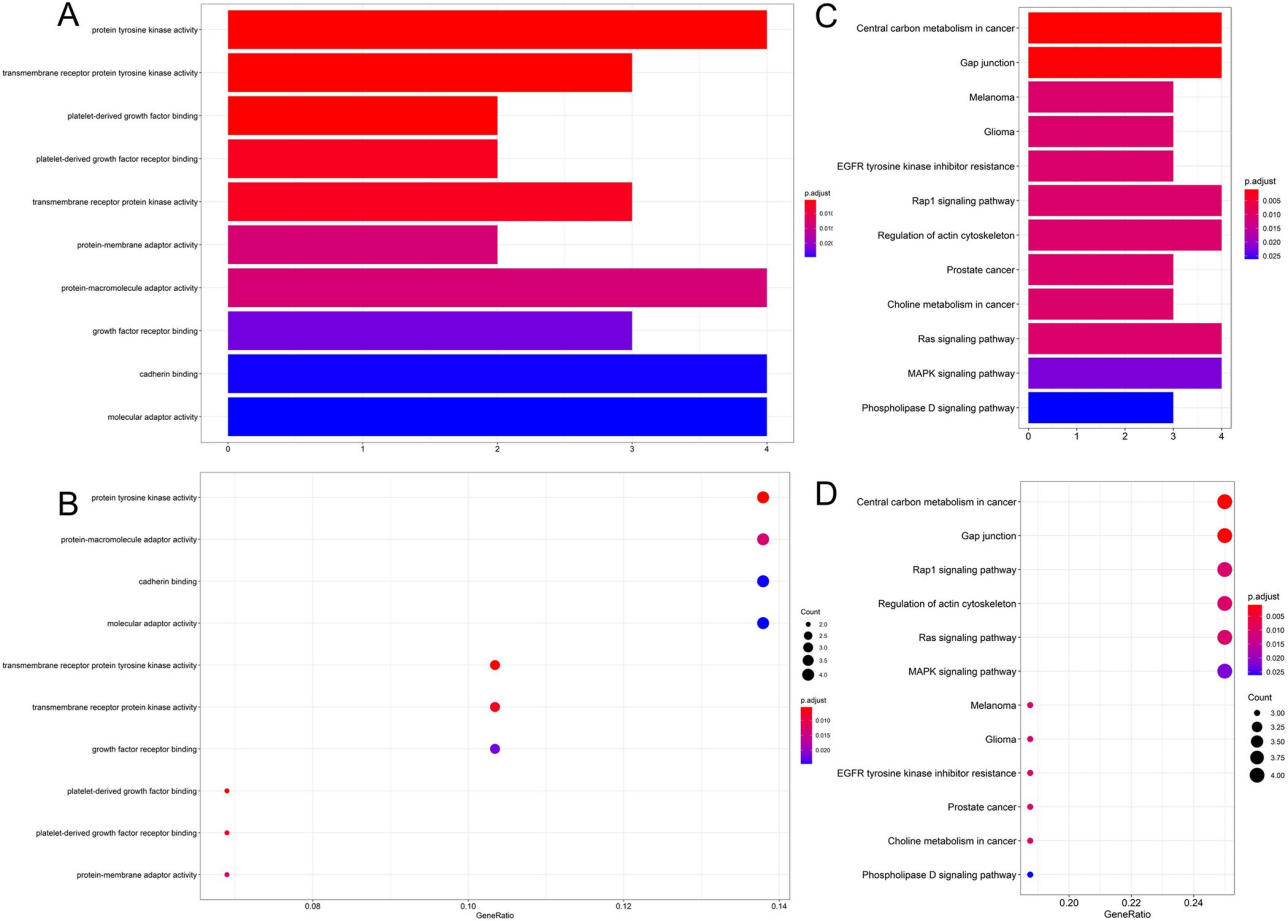
GO and KEGG enrichment analysis. A and B, GO enrichment analysis; C and D, KEGG enrichment analysis.

GO analysis revealed that the top five enriched mRNAs in the ceRNA network primarily focused on protein tyrosine kinase activity, 1-acyl-2-lysophosphatidylserine acylhydrolase activity, transmembrane receptor protein tyrosine kinase activity, and platelet-derived growth factor binding (Table 1). KEGG pathway analysis involving the ceRNA network revealed that Top7, which was predominantly enriched in target genes, was associated with central carbon metabolism in cancer, gap junction, Melanoma, Glioma, EGFR tyrosine kinase inhibitor resistance, Rap1 signaling pathway, and actin cytoskeleton regulation (Table 2). According to these findings, HCM occurrence and progression may involve various pathways and processes (Fig 5).

**Table 1:** GO enrichment analysis.

ID	Description	GeneRatio	BgRatio	pvalue	p.adjust	qvalue	geneID	Count
GO:0004713	protein tyrosine kinase activity	4/29	135/18337	5.78E-05	0.005529645	0.003557082	PDGFRB/PDGFRA/MAP2K1/CSF1R	4
GO:0004714	transmembrane receptor protein tyrosine kinase activity	3/29	61/18337	0.000120338	0.005529645	0.003557082	PDGFRB/PDGFRA/CSF1R	3
GO:0048407	platelet-derived growth factor binding	2/29	11/18337	0.000131658	0.005529645	0.003557082	PDGFRB/PDGFRA	2
GO:0005161	platelet-derived growth factor receptor binding	2/29	15/18337	0.000250363	0.006784512	0.004364306	PDGFRB/PDGFRA	2
GO:0019199	transmembrane receptor protein kinase activity	3/29	80/18337	0.000269227	0.006784512	0.004364306	PDGFRB/PDGFRA/CSF1R	3
GO:0043495	protein-membrane adaptor activity	2/29	24/18337	0.000652313	0.013332202	0.00857627	MYH9/SLC9A3R1	2
GO:0030674	protein-macromolecule adaptor activity	4/29	263/18337	0.000740678	0.013332202	0.00857627	MYH9/SLC9A3R1/FRMD4A/MAP2K1	4
GO:0070851	growth factor receptor binding	3/29	141/18337	0.001404636	0.022123018	0.014231181	SLC9A3R1/PDGFRB/PDGFRA	3
GO:0045296	cadherin binding	4/29	332/18337	0.001752875	0.024540255	0.015786129	MYH9/LDHA/CSNK1D/PTPRM	4
GO:0060090	molecular adaptor activity	4/29	342/18337	0.001953433	0.024613258	0.01583309	MYH9/SLC9A3R1/FRMD4A/MAP2K1	4

**Table 2:** KEGG enrichment analysis.

ID	Description	GeneRatio	BgRatio	pvalue	p.adjust	qvalue	geneID	Count
hsa05230	Central carbon metabolism in cancer	4/16	70/8086	8.67E-06	0.000962025	0.000748089	PDGFRB/LDHA/PDGFRA/MAP2K1	4
hsa04540	Gap junction	4/16	88/8086	2.16E-05	0.001197339	0.000931074	PDGFRB/CSNK1D/PDGFRA/MAP2K1	4
hsa05218	Melanoma	3/16	72/8086	0.000348857	0.010163902	0.007903651	PDGFRB/PDGFRA/MAP2K1	3
hsa05214	Glioma	3/16	75/8086	0.00039355	0.010163902	0.007903651	PDGFRB/PDGFRA/MAP2K1	3
hsa01521	EGFR tyrosine kinase inhibitor resistance	3/16	79/8086	0.000458666	0.010163902	0.007903651	PDGFRB/PDGFRA/MAP2K1	3
hsa04015	Rap1 signaling pathway	4/16	210/8086	0.000629442	0.010163902	0.007903651	PDGFRB/PDGFRA/MAP2K1/CSF1R	4
hsa04810	Regulation of actin cytoskeleton	4/16	218/8086	0.000724755	0.010163902	0.007903651	MYH9/PDGFRB/PDGFRA/MAP2K1	4
hsa05215	Prostate cancer	3/6	97/8086	0.000836763	0.010163902	0.007903651	PDGFRB/PDGFRA/MAP2K1	3
hsa05231	Choline metabolism in cancer	3/16	98/8086	0.000862144	0.010163902	0.007903651	PDGFRB/PDGFRA/MAP2K1	3
hsa04014	Ras signaling pathway	4/16	232/8086	0.000915667	0.010163902	0.007903651	PDGFRB/PDGFRA/MAP2K1/CSF1R	4
hsa04010	MAPK signaling pathway	4/16	294/8086	0.002203634	0.022236667	0.017291671	PDGFRB/PDGFRA/MAP2K1/CSF1R	4
hsa04072	Phospholipase D signaling pathway	3/16	148/8086	0.002824777	0.02612919	0.020318573	PDGFRB/PDGFRA/MAP2K1	3
hsa04151	PI3K-Akt signaling pathway	4/16	354/8086	0.004323212	0.036913579	0.028704727	PDGFRB/PDGFRA/MAP2K1/CSF1R	4

### Validation of cell hypertrophy model by WB

**Fig 6:**
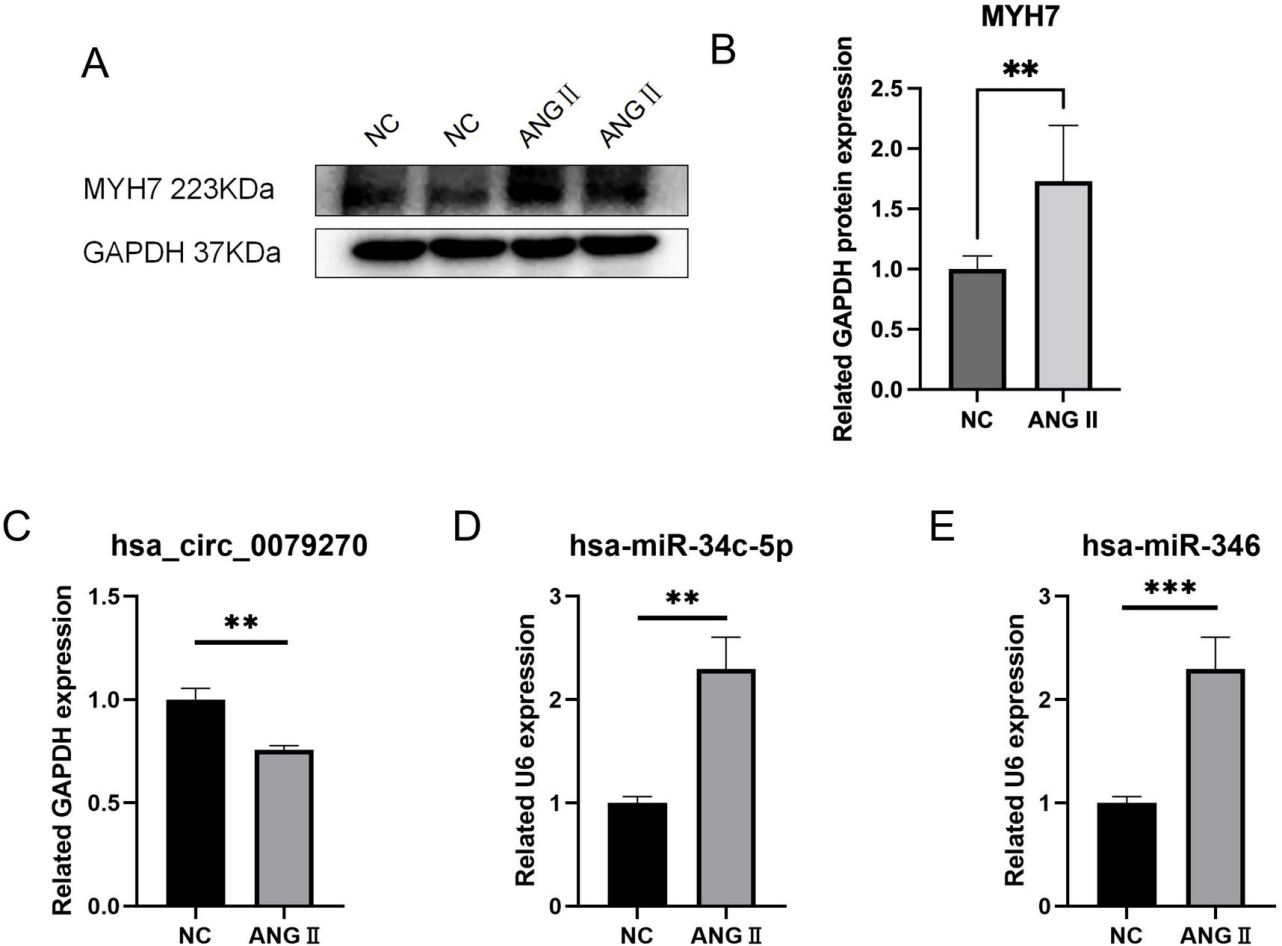
Experiment to verify potential targets. A and B, changes in the expression of MYH7 protein after ANGⅡ treatment. C, The RNA expression level of hsa_circ_0079270 in the cell hypertrophy model. D, the RNA expression of hsa-miR-34c-5p in the cell hypertrophy model. E, the RNA expression of hsa-miR-346 in the cell hypertrophy model. *<0.05, **<0.01, ***<0.001, ****<0.0001.

To validate the successful establishment of a cellular hypertrophy model, the expression of MYH7 protein (a conventional indicator protein of myocardial hypertrophy) in cells was confirmed by WB. The average MYH7 protein expression was 1.73 times higher in cells with hypertrophy than in cells from the control group (P = 0.0037), a statistically significant difference (Fig 6A and 6B and S5 File).

### qPCR verification of hub genes

**Fig 7:**
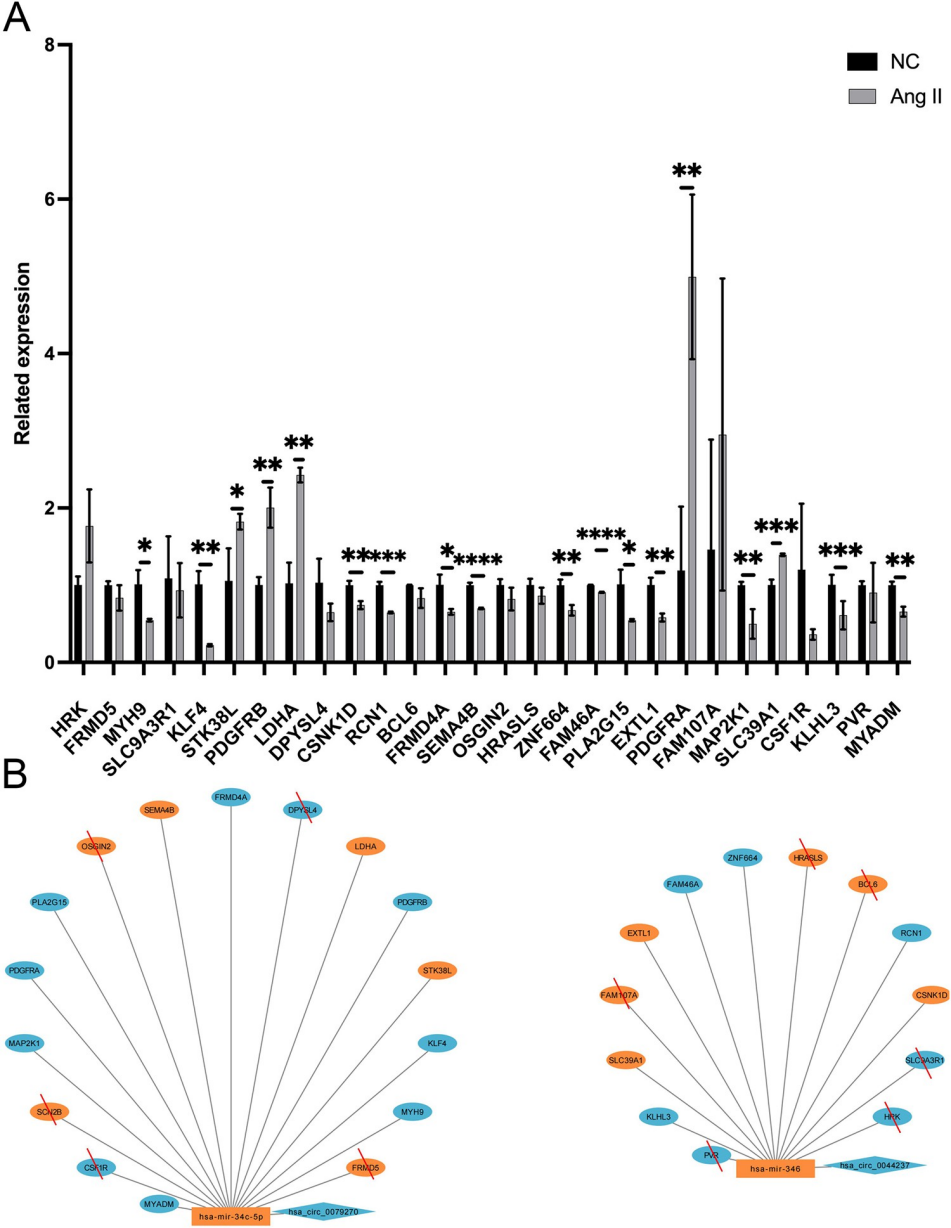
The RNA expression of mRNAs in the cell hypertrophy model and the modified ceRNA network diagram. A, Expression of mRNA in the cellular hypertrophy model compared to the control group. *<0.05, **<0.01, ***<0.001, ****<0.0001. B, The modified ceRNA network diagram. The results marked with red lines are negative results verified by the experiment, and those not marked are positive results.

**Fig 8:**
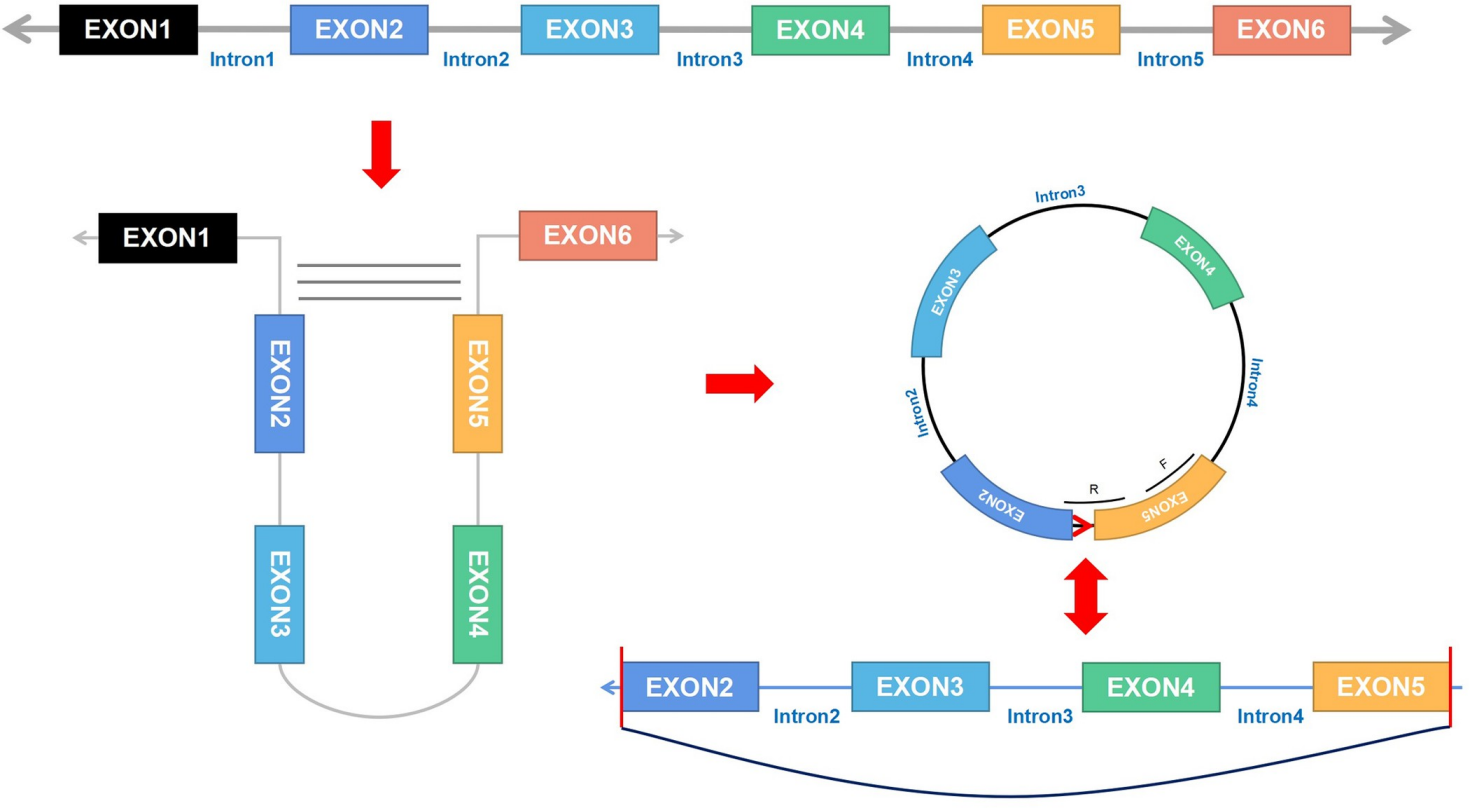
Schematic diagram of the transcription process of hsa_circ_0079270. During transcription, ligation is performed from before exon 2 and after exon 5 to form a circular RNA. F represents the forward primer and R represents the reverse primer.

qPCR was employed to examine the expression of three genes (including one circRNA and two miRNAs) in AC16 cell lines. Consistent with evidence from bioinformatics analyses, in cell-cell hypertrophy, the RNA expression of hsa circ 0079270 was much lower than that in NC, with a 0.76-fold average decrease (P = 0.0047) (Fig 6C). Hsa-miR-34c-5p was upregulated in hypertrophic cells, with a 2.28-fold average increase (P = 0.0020); hsa-miR-346 RNA expression was 2.82 times higher than that of NC (P = 0.0002), with statistically significant differences. (Fig 6D and 6E) The RNA expression levels of MYH9, KLF4, STK38L, PDGFRB, LDHA, CSNK1D, RCN1, FRMD4A, SEMA4B, ZNF664, FAM46A, PLA2G15, EXTL1, PDGFRA, MAP2K1, SLC39A1, KLHL3, and MYADM were statistically different (P<0.05) (Fig 7). Hsa circ 0079270 is the result of reverse transcription and partial splicing of the ACTB DNA sequence, joining exons 2 and 5 to form a circular shape (Fig 8).

## Discussion

CircRNAs are single-stranded RNAs with no free ends that form a covalently closed loop. Numerous circRNAs are endogenous, stable, and abundant, with cell type-, tissue-, and developmental-stage-specific expression patterns in eukaryotic cells. In recent years, the rapid development of biochemical techniques and the application of high-throughput sequencing technology have made it feasible to isolate and identify a greater variety of circRNAs. Several studies have demonstrated that circRNAs are associated with numerous physiological and pathological processes [16, 17, 24].

Cardiovascular disease is one of the leading causes of death globally. Recent studies have demonstrated that circRNAs are associated with numerous cardiovascular disorders. The effect of circRNAs on the cardiovascular system is unclear. A deeper understanding of circRNAs will lay the groundwork for diagnostic and therapeutic strategy development for cardiovascular disease [14]. As stable, abundant, and conserved ceRNAs that act as miRNA sponges, circRNAs could be useful markers for diagnosing HCM and evaluating its etiology [25, 26]. Similar findings corroborated the hypothesis that the DE circRNAs found in our investigation may be essential components of the ceRNA network, which modulates essential gene expression in the onset and progression of cardiovascular illnesses, particularly HCM. HCM is one of the most prevalent genetic heart illnesses and is associated with an elevated risk of sudden cardiac death [9]. Significant efforts have been made over the past few decades to understand the molecular mechanism of HCM, with a focus on protein-coding genes, the majority of which are responsible for sarcomere formation [27]. Recently, it has been widely reported that circRNAs are involved in a vast array of biological processes and that their dysregulated expression is associated with several complicated human disease phenotypes, including cardiovascular disorders. These findings suggest that circRNAs may play a role in the progression of HCM and may serve as crucial indicators for diagnosis and treatment targeting [15, 24].

Currently, there is little research on hypertrophy and circRNA. RNA‐Seq data also showed significant differences in circRNA expression profiles in dilated cardiomyopathy (DCM) and HCM hearts compared with normal control hearts [28]. In particular, circRNAs produced by CAMK2D genes (in DCM and HCM) and titin genes (in DCM) were reduced. Qi et al. performed circRNA sequencing on HCM patient samples for the first time in a recent study and received extremely valuable data [29]. Using WGCNA, researchers determined that circRNAs hsa circ 0043762, hsa circ 0036248, and circ 0071269 may serve as possible regulators of HCM. Additionally, circRNAs and HCM were the subjects of this study. Several circRNA expression patterns were investigated as possible indicators of HCM by Sonnenschein et al. [30]. This study enrolled 64 patients with HCM and 53 healthy controls. The quantitative expression of a collection of circRNAs known to be associated with heart disease was assessed in blood. In another study on animal models of myocardial hypertrophy, 3 mRNA, 4 miRNAs, and 4 circRNAs were found to play an important role in myocardial hypertrophy. Bioinformatics methods were used for the study [31].

In a TAC-induced mouse model of cardiomyopathy induced by pressure overload, high circ Foxo3 expression was found to be involved in the protective mechanism of Ganoderma spore oil against cardiomyopathy [32]. In another study, we found that ganoderma spore oil improved cardiac function and reduced elevated circ‐Foxo3 expression in doxorubicin (Dox) DOX-damaged hearts [33]. Recently, Zeng et al. demonstrated the role of circ‐Amotl1, derived from the angiopoietin-like 1 gene (Amotl1), in DoX-induced animal cardiomyopathy [34]. Intraperitoneal injection of circ‐Amotl1 alleviated the abnormal effects of Dox on the heart, which was characterized by reduced apoptosis, hypertrophy, and fibrosis. These findings highlight the potential of circRNAs as a therapeutic intervention for cardiomyopathy. However, regardless of whether it is a basic experiment or a data analysis, the results of many experiments and analyses may be different or even contradictory. This may be due to the small sample size, different races collected, and the use of different algorithms and detection methods, leading to inconsistent results. However, these study aims to lay the groundwork for research in this area despite the small sample size. With more studies with increased sample sizes, the results of these preliminary studies can provide potential screening targets for future studies. This finding is of great significance for future research on the significance of circRNAs in HCM.

This investigation examined the overlap between HCM-specific DE circRNAs, DE miRNAs, and DE mRNAs in the GEO database and circRNA and miRNA target molecules predicted by related databases. Two circRNAs, two miRNAs, and thirty mRNAs were found to establish a circRNA-miRNA-mRNA regulatory network, which may play a significant role in the progression of HCM. Subsequently, we performed GO and KEGG pathway analyses on the 30 mRNAs to increase our understanding of the crucial pathophysiological mechanisms underlying the onset and progression of HCM. The majority of the two DE circRNAs found in our analysis were novel biomarkers for HCM that require further investigation.

GO enrichment was primarily concentrated on protein tyrosine kinase activity, 1-acyl-2-lysophosphatidylserine acylhydrolase activity, transmembrane receptor protein tyrosine kinase activity, platelet-derived growth factor binding, and phosphatidylserine 1-acylhydrolase activity. These two circRNAs exhibited high levels of calcium release channel activity. Since disturbance of calcium homeostasis is one of the most prevalent causes, mutation-specific alterations in the rate of calcium release in HCM are closely correlated with the disease [35–39]. Additionally, calcium homeostasis abnormalities may worsen diastolic dysfunction, resulting in heart failure and substantial morbidity and mortality [40–42]. Platelet-derived growth factor binding is intimately associated with cardiomyocyte fibrosis and is also one of the mechanisms underlying HCM [43]. Additionally, KEGG research demonstrated that it may be associated with gap junctions, EGFR tyrosine kinase inhibitor resistance, Rap1 signaling pathway, and regulation of the actin cytoskeleton. These mechanisms are similar to the calcium release channel activity described previously [44–48]. This strongly suggests that the circRNAs found in the ceRNA network may play an essential role in the etiology of HCM.

In this study, AC16 cells from the human heart were used to develop an angiotensin II-based model of cardiac hypertrophy. MYH7 was employed as an indicator protein to validate the model’s construction by detecting differences in its expression using WB. The total RNA of the cell hypertrophy model was extracted, and the circRNAs and miRNAs identified by qPCR were confirmed. It has been discovered that there is differential expression between one circRNA, two miRNAs, and eighteen mRNAs. These results demonstrated that these hub genes regulate cardiomyocyte hypertrophy.

Although altered circRNAs, miRNAs, and mRNA have been found and their potential relevance in the pathophysiology of HCM has been investigated, certain limitations must be taken into account when interpreting our results. Due to the lack of accessible data, the strength of the statistical findings may be limited, and the strength of any subtype analysis may be weak. Multiple databases were utilized to anticipate the interactions between circRNAs and miRNAs and between mRNAs and miRNAs to ensure their consistency and reliability. With the introduction of larger sample sizes, improved databases, and improved algorithms, a more comprehensive ceRNA network could be constructed in the future. To verify the qPCR experiments, hsa circ 0044237 generated three pairs of primers; however, the results of these primers were too low to examine. Hence, this circRNA was not present in our analysis results. Additionally, molecular biology techniques, including qPCR, luciferase reporter system, and immunoprecipitation analysis, may help validate our findings and reveal the molecular process behind the ceRNA network in HCM.

## Conclusion

Our study established a network of circRNA-related ceRNAs in HCM. Network-identified circRNAs may be the most important risk factors for HCM etiology. From the standpoint of the circRNA-miRNA-mRNA network, our study provides novel insights into the pathophysiology of HCM.

## Supporting information

S1 File mRNA primer sequence. (ZIP)

S2 File Raw data. (ZIP)

S3 File qPCR experimental data. (XLSX)

S4 File The program code of the whole experiment. (ZIP)

S5 File Raw images. (ZIP)
