# Supplementary figures and images for: Identification of circRNA-miRNA-mRNA regulatory network and its role in cardiac hypertrophy
Source: PLoS One. 2023 Mar 23;18(3):e0279638. doi: 10.1371/journal.pone.0279638 (PMC10035836; doi:10.1371/journal.pone.0279638)

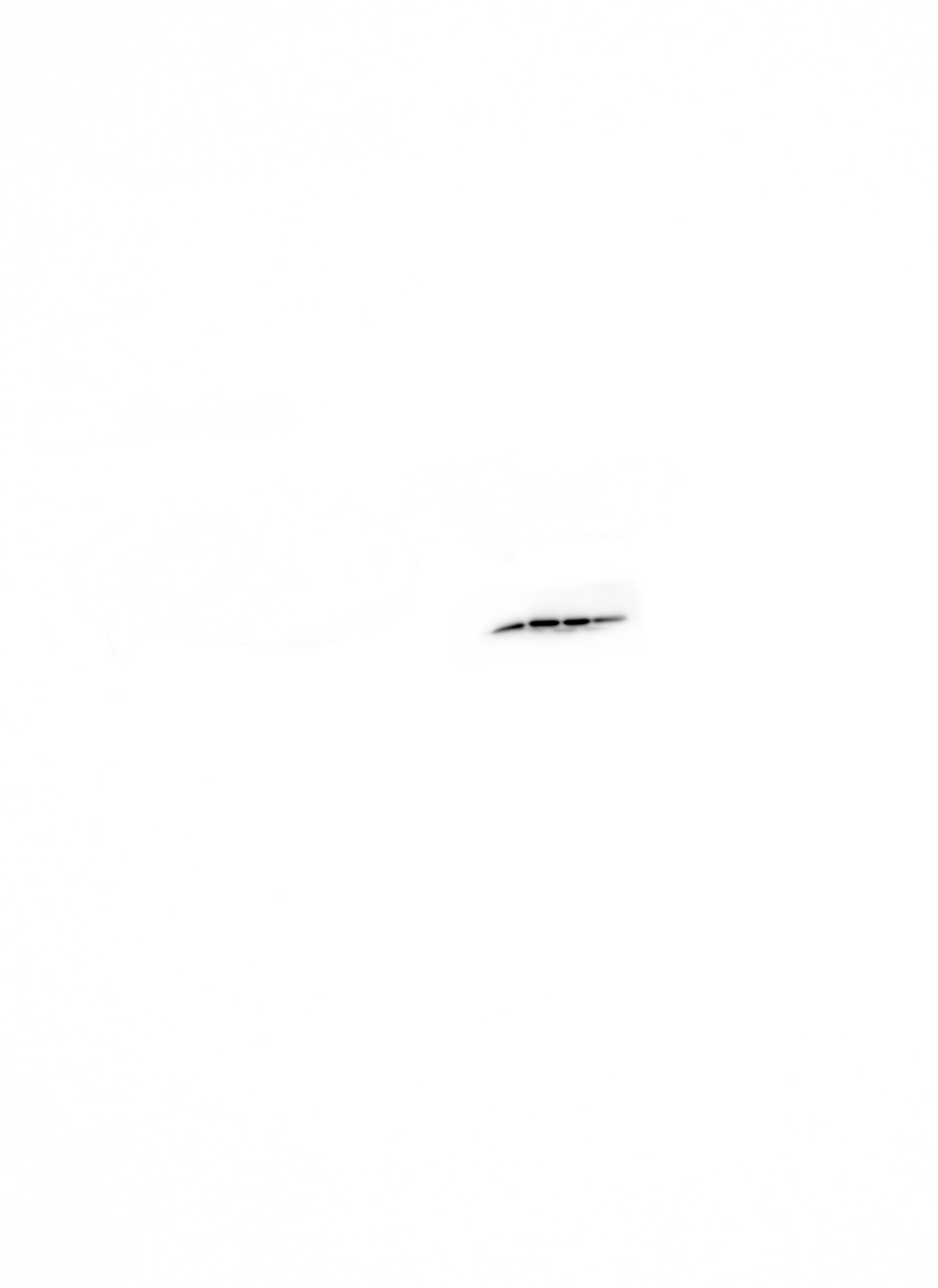

Supplement: S5 File — (ZIP) [file pone.0279638.s005.zip › S5/gapdh1.jpg]

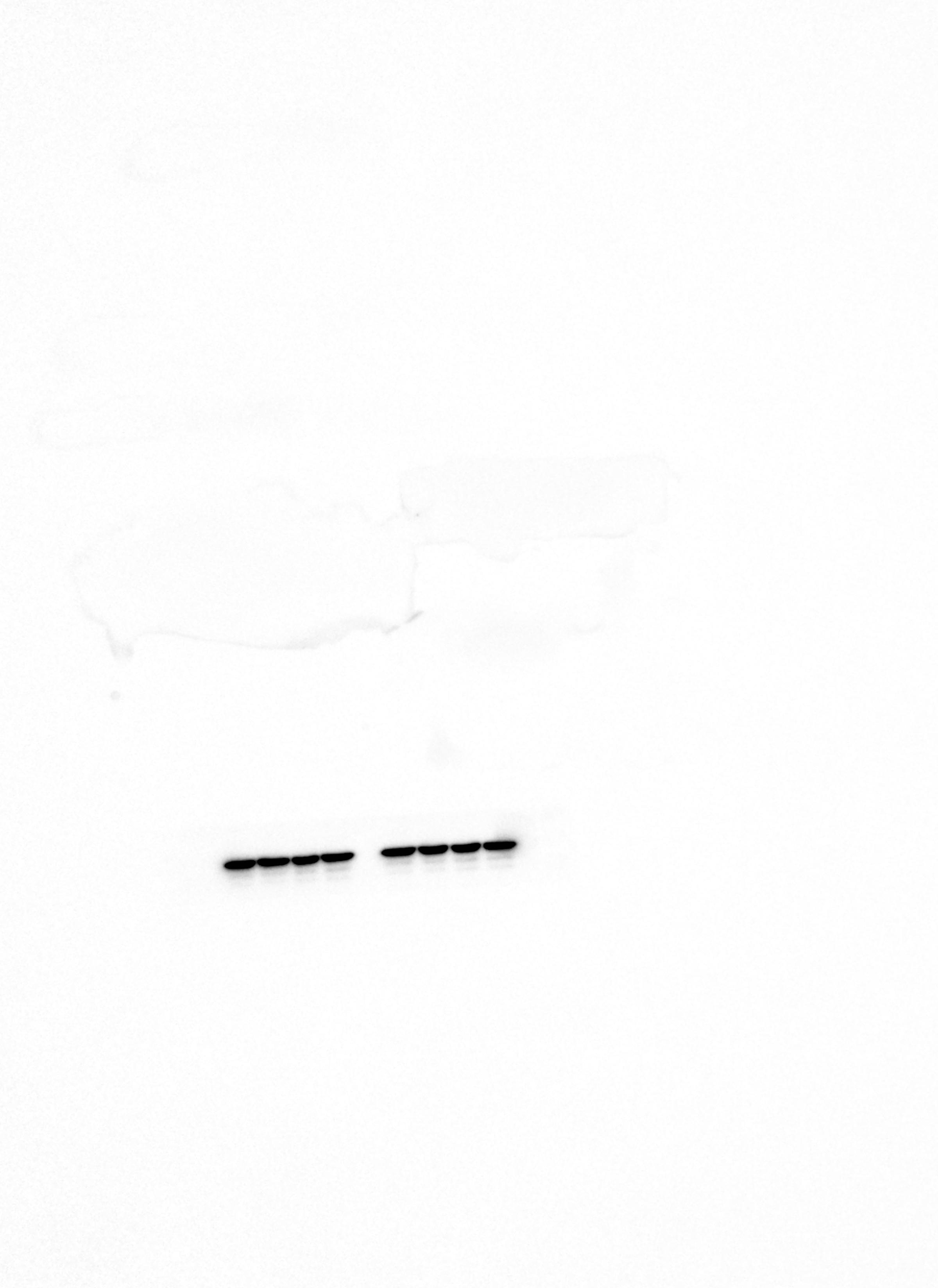

Supplement: S5 File — (ZIP) [file pone.0279638.s005.zip › S5/gapdh 2.jpg]

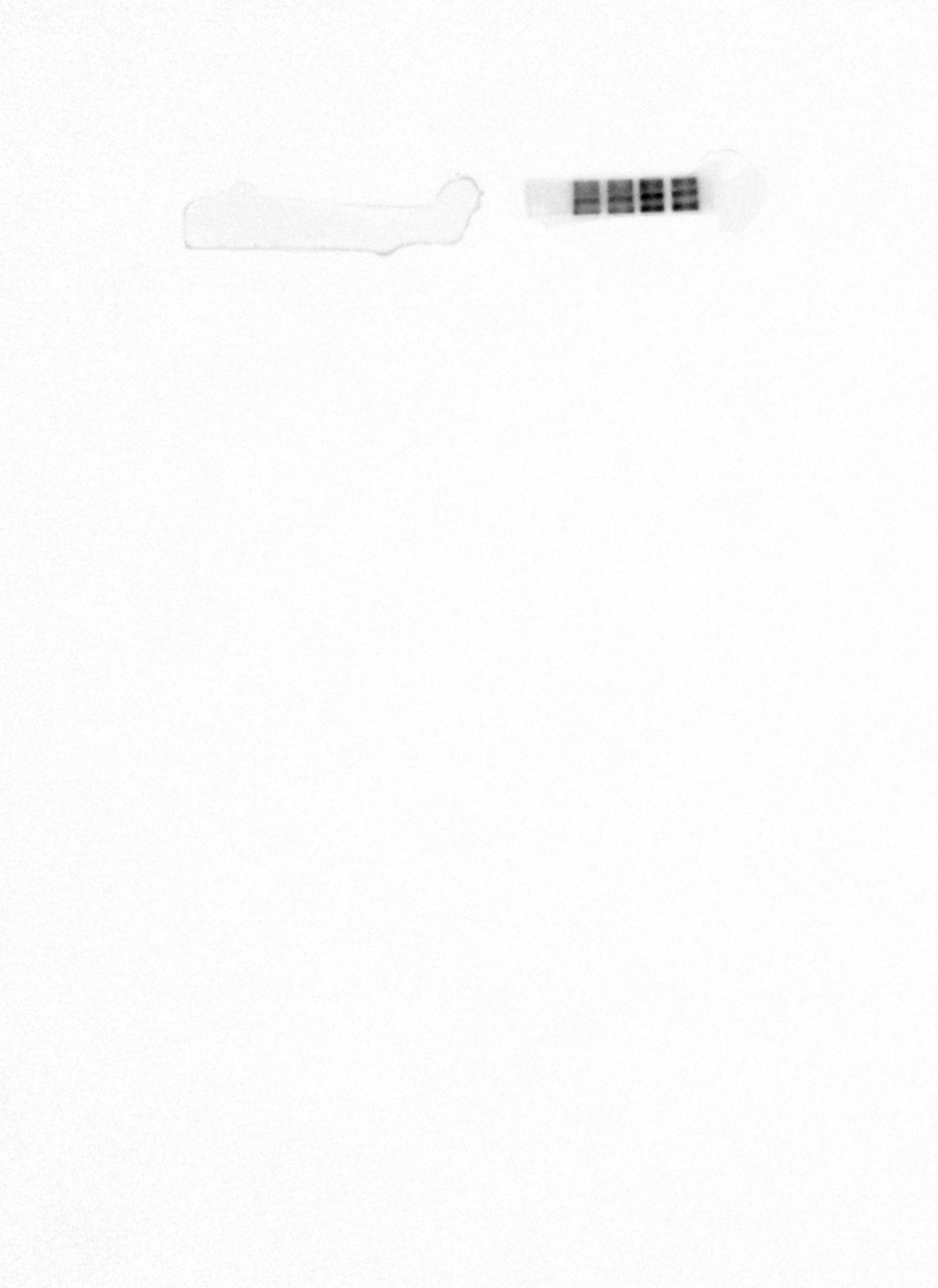

Supplement: S5 File — (ZIP) [file pone.0279638.s005.zip › S5/myh71.jpg]

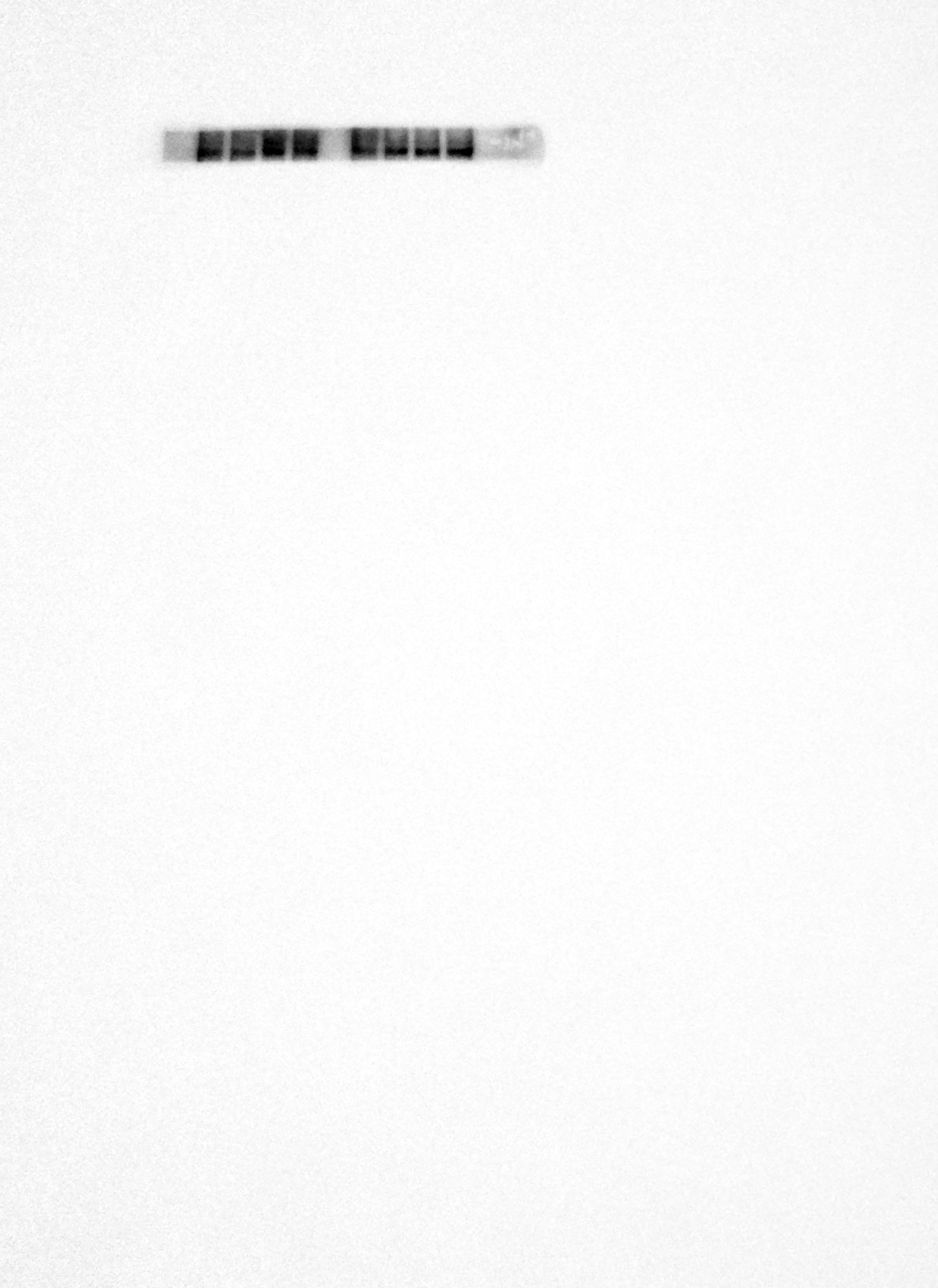

Supplement: S5 File — (ZIP) [file pone.0279638.s005.zip › S5/myh72.jpg]

Raw data of WB


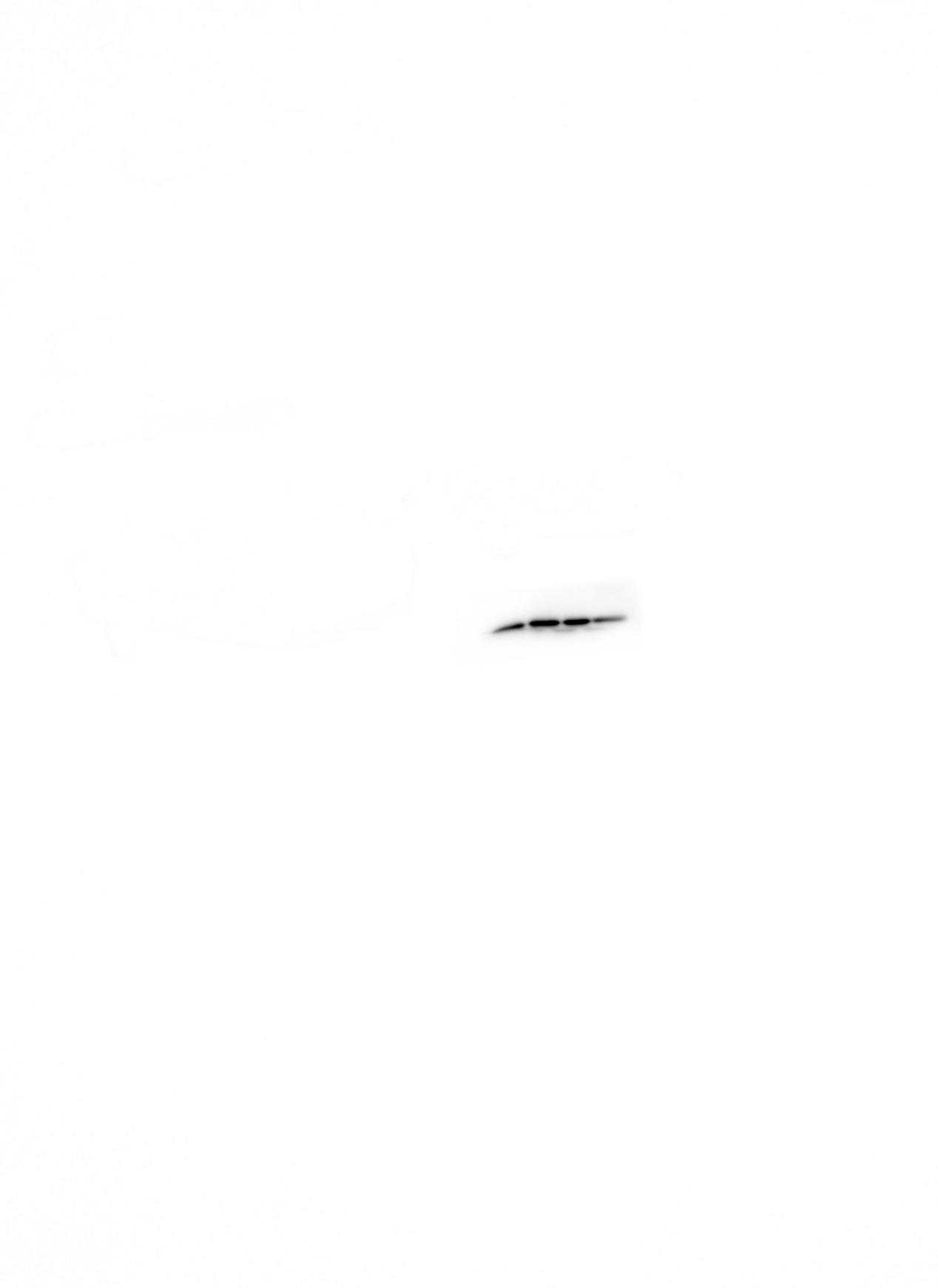

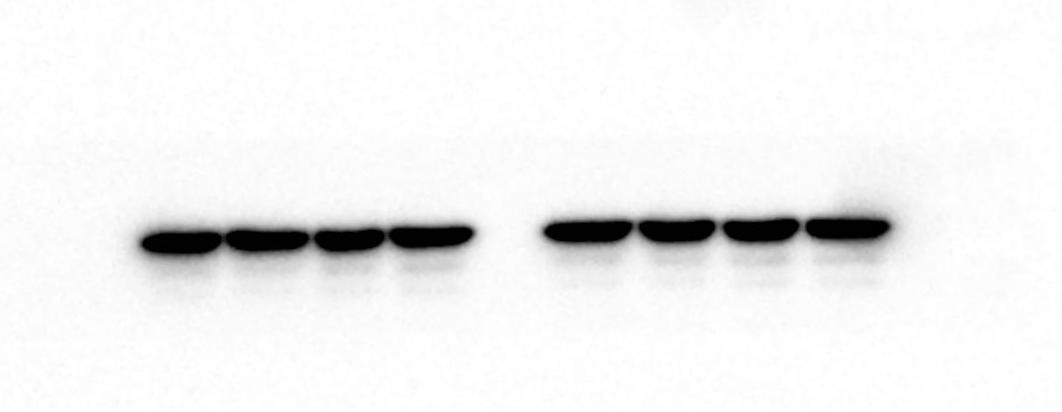

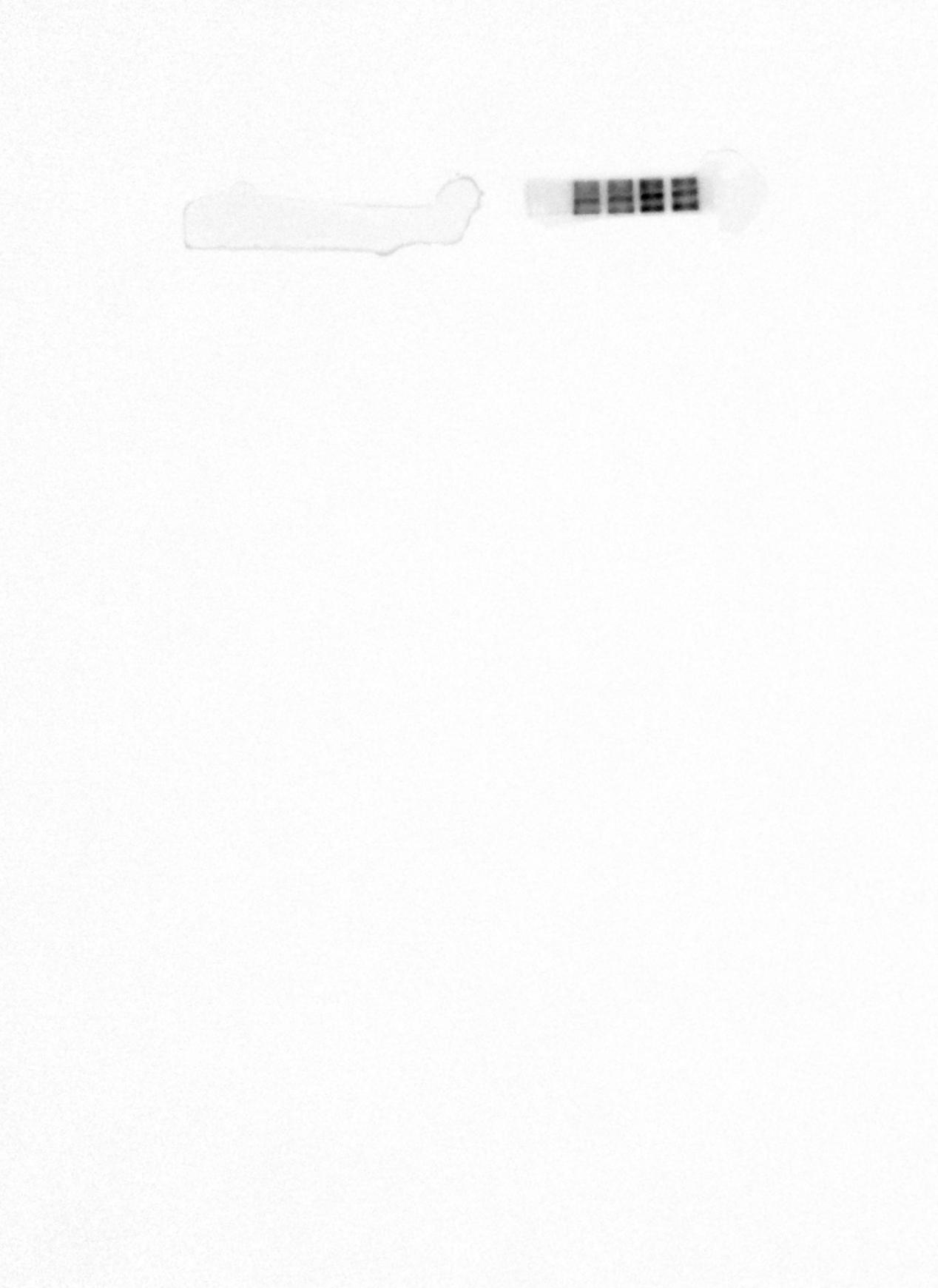

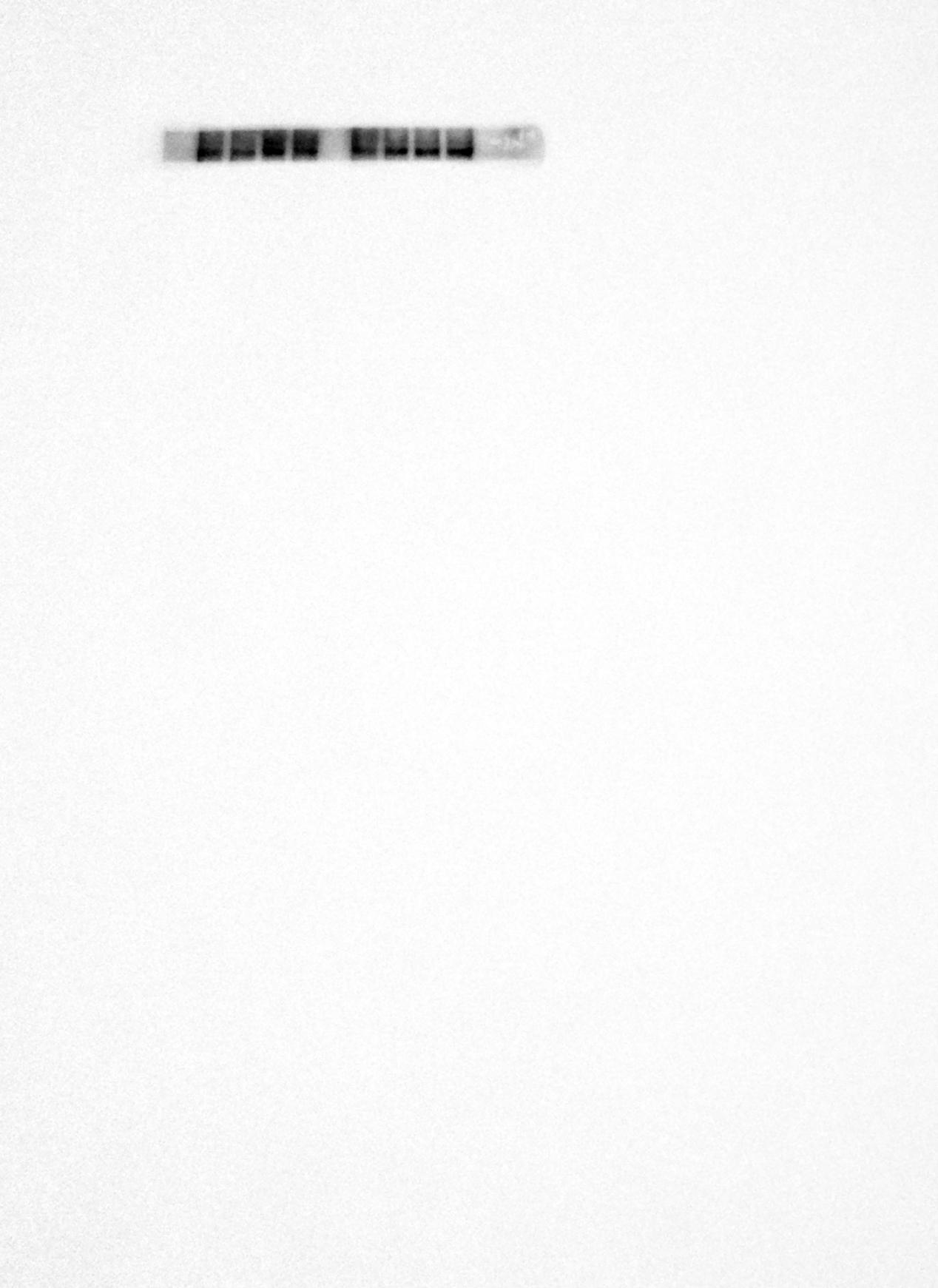

Supplement: S5 File — (ZIP) [file pone.0279638.s005.zip › S5/Raw data of WB.docx]

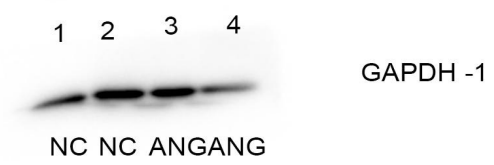

Amersham ImageQuant™ 800

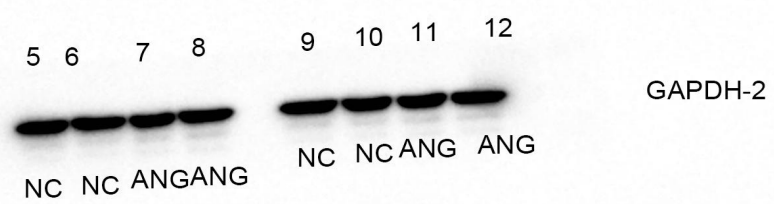

Amersham ImageQuant™ 800

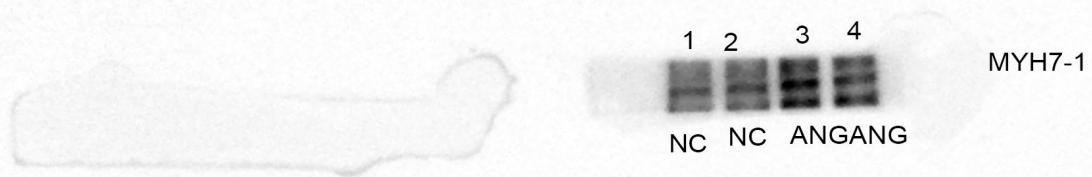

Amersham ImageQuant™ 800

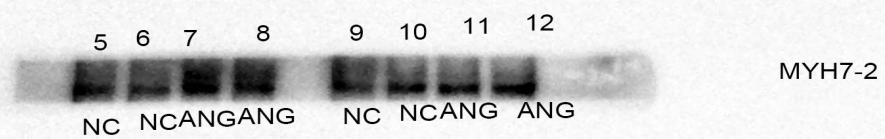

Amersham ImageQuant™ 800

Supplement: S5 File — (ZIP) [file pone.0279638.s005.zip › S5/S5_Raw_images.pdf]
